# Salvia-Nelumbinis naturalis improves lipid metabolism of NAFLD by regulating the SIRT1/AMPK signaling pathway

**DOI:** 10.1186/s12906-022-03697-9

**Published:** 2022-08-09

**Authors:** Yang Liu, Yiping Li, Jue Wang, Lili Yang, Xiao Yu, Ping Huang, Haiyan Song, Peiyong Zheng

**Affiliations:** 1 Institute of Digestive Diseases, Longhua Hospital, Shanghai University of Traditional Chinese Medicine, Shanghai, 200032 China; 2 Teaching Experiment Center, Shanghai University of Traditional Chinese Medicine, Shanghai, 201203 China; 3 Department II of Digestive Diseases, Longhua Hospital, Shanghai University of Traditional Chinese Medicine, Shanghai, 200120 China

**Keywords:** Nonalcoholic fatty liver disease, Salvia-Nelumbinis naturalis, SIRT1, AMPK, Lipid metabolism

## Abstract

**Background:**

Salvia-Nelumbinis naturalis (SNN), the extract of Chinese herbal medicine, has shown effects on NAFLD. This study aims to explore the underlying mechanism of SNN for regulating the lipid metabolism disorder in NAFLD based on the SIRT1/AMPK signaling pathway.

**Methods:**

Male C57BL/6J mice fed with a high-fat diet (HFD) were used to establish the NAFLD model. Dynamic changes of mice including body weight, liver weight, serological biochemical indexes, liver histopathological changes, and protein level of AMPK and SIRT1 were monitored. After18 weeks, SNN treatment was administrated to the NAFLD mice for another 4 weeks. Besides the aforementioned indices, TC and TG of liver tissues were also measured. Western blot and quantitative RT-PCR were used to detect the expression and/or activation of SIRT1 and AMPK, as well as the molecules associated with lipid synthesis and β-oxidation. Furthermore, AML12 cells with lipid accumulation induced by fatty acids were treated with LZG and EX527 (SIRT1 inhibitor) or Compound C (AMPK inhibitor ) to confirm the potential pharmacological mechanism.

**Results:**

Dynamic observation found the mice induced by HFD with gradually increased body and liver weight, elevated serum cholesterol, hepatic lipid accumulation, and liver injury. After 16 weeks, these indicators have shown obvious changes. Additionally, the hepatic level of SIRT1 and AMPK activation was identified gradually decreased with NAFLD progress. The mice with SNN administration had lower body weight, liver weight, and serum level of LDL-c and ALT than those of the NAFLD model. Hepatosteatosis and hepatic TG content in the liver tissues of the SNN group were significantly reduced. When compared with control mice, the NAFLD mice had significantly decreased hepatic expression of SIRT1, p-AMPK, p-ACC, ACOX1, and increased total Acetylated-lysine, SUV39H2, and SREBP-1c. The administration of SNN reversed the expression of these molecules. *In vitro* experiments showed the effect of SNN in ameliorating hepatosteatosis and regulating the expression of lipid metabolism-related genes in AML12 cells, which were diminished by EX527 or Compound C co-incubation.

**Conclusions:**

Taken together, the SIRT1/AMPK signaling pathway, involved in hepatic lipid synthesis and degradation, plays a pivotal role in the pathogenesis of NAFLD development. The regulation of SIRT1/AMPK signaling greatly contributes to the underlying therapeutic mechanism of SNN for NAFLD.

**Supplementary Information:**

The online version contains supplementary material available at 10.1186/s12906-022-03697-9.

## Background

Nonalcoholic fatty liver disease (NAFLD) is the liver injury induced by metabolic stress, which is highly correlated with insulin resistance, oxidative stress, and genetic susceptibility. This disease ranges from nonalcoholic fatty liver (NAFL) with simple fatty infiltration of the liver to nonalcoholic steatohepatitis (NASH) with inflammation and hepatocyte ballooning in addition to diffuse steatosis, eventually leading to cirrhosis and hepatocellular carcinoma (HCC) [1]. In recent years, the prevalence of NAFLD increased quickly, afflicting around 25% of the population worldwide. Approximately 20% -30% of NAFL patients could develop into NASH, and 25% of them may further progress to cirrhosis [2].

The pathogenesis of NAFLD is complicated and has not been completely clear by far. Excess fatty acids generated in the liver or fluxed from adipose lipolysis, if their disposal by oxidation or release of VLDL is impaired or overwhelmed, can generate lipotoxic lipid species. Such lipids then cause mitochondria dysfunction, endoplasmic reticulum stress, inflammation, apoptosis, and activation of stellate cells. Thus dysfunctional lipid metabolism triggers subsequent liver injury [3, 4]. Recent studies have shown that AMP-activated protein kinase (AMPK) and Sirtuin1 (SIRT1) are the key enzymes responsible for energy homeostasis by regulating fatty acid metabolism [5, 6]. AMPK functions as an energy switch in regulating the synthesis and β-oxidation of fatty acids by regulating the associated gene expression and activation [7]. SIRT1 is an NAD^+^-dependent protein deacetylase, which functions as an important regulator of energy hemostasis and lipid metabolism in mammals [8]. SIRT1 activity relies on AMPK and was also shown to regulate AMPK activation in NAFLD [9, 10].

Lifestyle changes have shown benefit for NAFLD. However, it is challenging to sustain in a long term critically, and currently, there are no approved efficient drugs for the treatment of NAFLD and NASH [11]. In recent years, traditional Chinese medicine (TCM) has displayed effects in the treatment of NAFLD. Salvia–Nelumbinis naturalis (SNN) is the extract of a compound formula of Chinese medicine (also named Jiang Zhi Granule), composed of five medicinal herbs, namely, *Salvia miltiorrhiza* Bunge., *Nelumbo nucifera* Gaertn., *Gynostemma pentaphyllum* (Thunb.) Makino*, Polygonum cuspidatum* Sieb. *et* Zucc., and *Artemisia capillaris* Thunb. It has been used to treat NAFLD in China, with significant effects of alleviating hepatic steatosis with few side effects [12]. Previous *in vivo* studies have confirmed the effect of SNN on NAFLD, alleviating high-fat diet (HFD)-induced or methionine-choline-deficiency (MCD) diet-induced hepatosteatosis and lipotoxic liver injury as well as serum levels of transaminases and lipid in male C57BL/6 mice or SD rats [13–16]. *In vitro* studies have also indicated that SNN could reduce lipid droplet accumulation and increase the resistance to damage of hepatocytes induced by free fatty acids [17, 18]. Moreover, the underlying mechanisms were partially unraveled, including improving insulin resistance, enhancing autophagy and anti-oxidative stress, and inhibiting transcription of liver X receptor α (LXR-α)-mediated sterol regulatory element binding protein-1c (SREBP-1c), et al [13, 15–17].

The pathophysiology of NAFLD is regarded to be constituted by multiple parallel hits, leading to an imbalance of energy metabolism in the liver, mostly in the form of carbohydrates and fat [4, 19]. The compound formulae of TCM usually have the advantage of multi-target effects. Therefore, in the present study, through the intervention experiment of the HFD-induced NAFLD mice model and fatty acid-induced hepatocytes with SNN, we tried to investigate the mechanism of SNN based on the lipid metabolism-regulating pathway, SIRT1/AMPK. This will help in understanding the therapeutic effect of the traditional herbal formula and promoting its further clinical application.

## Materials and Methods

### Experimental animals and drugs

Male C57BL/6J mice of 6-week-old (SLAC Laboratory Animal Technology Company, Shanghai, China) were used in the animal experiment. The mice were housed in a standard 12 h light/dark cycle at 22 ± 2°C with 55 ±10% humidity. The procedures of animal experiments in this study were carried out according to the guideline for the care and use of laboratory animals and approved by the Institutional Animal Care and Use Committee of Longhua Hospital, Shanghai University of Traditional Chinese Medicine (IACUC approval number.: LHERAW-2019001). This study is reported in accordance with ARRIVE guidelines (https://arriveguidelines.org).

SNN was provided by the Department of Pharmacy of Longhua Hospital, Shanghai. The composition is listed in Table 1. All of the raw herbal medicines in the formula were blended and reflux extracted by water, which was subsequently concentrated, and extracted with ethanol as described before. The chemical profile has been previously analyzed by ultra-performance liquid chromatography [20].

**Table 1:** The composition of Salvia-Nelumbinis naturalis (SNN)

Herbal name	Medicinal part	Proportion
*Salvia miltiorrhiza* Bunge.	Root	1.5
*Nelumbo nucifera* Gaertn.	Leaf	1
*Gynostemma pentaphyllum* (Thunb.) Makino	Herb	2.5
*Polygonum cuspidatum* Sieb. *et* Zucc.	Root and rhizome	2.5
*Artemisia capillaris* Thunb.	Aerial part	1.5

### Design of animal experiment

A total of 60 C57BL/6J mice were randomly assigned to the control group (*n* = 24) and model group (*n* = 36) according to body weights. The control mice were fed with a standard chow diet, the model mice were fed with a high-fat diet (HFD, 60% calories from fat) (ResearchDiets, Inc., NewBrunswick, NJ, USA). All the mice were fed with diet and water ad libitum. The dynamic change of serum aminotransferase, serum lipid, and hepatic histological changes was investigated after 4 weeks, 8 weeks, 12 weeks, and 16 weeks respectively (*n* = 3 for each group).

From the 19th week, the mice fed with the HFD diet were subdivided into the Model group and SNN group (*n* = 12 per group). SNN (860 mg/kg body weight /day) or an equal volume of normal saline were administrated by gavage to mice of the SNN group or model group respectively, together with HFD feeding. The dose of SNN was calculated according to previous experiments [15]. The left mice fed with a chow diet were still allocated to the Control group (*n* = 12). The food intake was recorded. At the end of the 22nd week, all animals were fasted overnight, anesthetized by intraperitoneal injection of 30 mg/kg pentobarbital sodium, and sacrificed. The serum and liver tissues were collected for further investigation.

### Serum biochemical analysis

The serum level of aspartate aminotransferase activity (AST), alanine aminotransferase activity (ALT), triglycerides (TG), total cholesterol (TC), high-density lipoprotein-cholesterol (HDL-c), and low-density lipoprotein-cholesterol (LDL-c) of the mice were detected using a biochemistry analysis system (Beckman AU5800 ) and corresponding reagent kits according to the manufacturer’s instructions in Laboratory Department of Longhua Hospital.

### Histological examination

The fresh liver tissues were fixed in 10% neutral formalin and embedded in paraffin, cut into 4μm slices, and stained with a series of hematoxylin and eosin (HE) staining solutions. Then the stained samples were photographed with a light microscope (Olympus, Tokyo, Japan). The Oil Red O staining of liver tissues was performed as described previously [15]. Briefly, the 10 μm-thick frozen liver sections were fixed with 10% paraformaldehyde for 30 min, then stained with Oil Red O solution and counterstained with diluted hematoxylin (1:10 ). The mounted stained sections were photographed under a light microscope.

To quantitatively assess the effect of SNN on the histopathological changes, NAFLD activity score (NAS) based on histological features were scored: steatosis (0–3), lobular inflammation (0–3), and hepatocellular ballooning (0–2) [21].

### Evaluation of lipid content of liver tissues

Hepatic tissues were homogenized with ethanol–acetone (1:1) in an ice bath and stayed at 4°C overnight. Subsequently, the homogenized tissues were centrifuged at 3000 rpm, 4°C for 20 min, and the supernatant was collected. The measurement of TG or TC content was performed according to the instructions of the assay kit (Jiancheng Institute of Bio Engineering Inc., Nanjing, China) by using the colorimetric method.

### Quantitative reverse transcription-polymerase chain reaction (qRT-PCR)

The total RNA of liver tissues or cells was extracted using TRIzol reagent solution (Invitrogen, Carlsbad, CA, USA). The gene expression was determined by qRT-PCR as described in our previous study [15]. The cDNA was synthesized using reverse transcription kits (Promega, Madison, WI, USA). The genes were amplified using an SYBR Green PCR Master Mix kit (Applied Biosystems, Carlsbad, CA, USA) and the specific primers (sequences are listed in Table 2). Amplification of β-actin was used as the internal control. The final data analysis was performed using the 2^−ΔΔCt^ method.

**Table 2:** The primer sequences for quantitative PCR used in this study

Gene	Primer sequence
β-actin	Forward: 5’-GCTGTCCCTGTATGCCTCTG-3’
	Reverse: 5’-GCTGTCCCTGTATGCCTCTG-3’
FASN	Forward: 5’-CCTGCCTCTGGTGCTTGC-3’
	Reverse:5’-GCCTCCTTGATATAATCCTTCTGA-3’
ACOX1	Forward: 5’-CTCGGAAGATACATAAAGGAGACC-3’
	Reverse:5’-CCAGGTAGTAAAAGCCTTCAGC-3’
SIRT1	Forward :5’-AAAGTGATGACGATGACAGAACG-3’
	Reverse :5’-GCCAATCATGAGATGTTGCTG-3’

### Western blot

Proteins were extracted using RIPA lysis buffer, then resolved by 10% denaturing SDS-PAGE and transferred onto PVDF membranes (Millipore, Billerica, MA, USA). The membranes were incubated with specific primary antibodies at 4°C overnight. The antibodies against AMPKα, Phosphorylated-AMPKα, Acetyl-CoA Carboxylase (ACC), and Phosphorylated-ACC were purchased from Cell Signaling Technology (Danvers, MA, USA). The antibodies against SIRT1 and SREBP-1c were obtained from Abcam (Cambridge, MA, USA) and antibodies against suppressor of variegation 39 homolog 2 (SUV39H2), fatty acid synthase (FASN), acyl-Coenzyme A oxidase (ACOX), and peroxisome proliferator activated receptor-α (PPAR-α) from Proteintech (Wuhan, China). Subsequently, the membranes were incubated with goat anti-rabbit or anti-mouse secondary antibodies (Thermo Scientific, Rockford, IL, USA) at room temperature for 1 h. Next, the enhanced chemiluminescence HRP substrate (Millipore) was added. The signal of protein bands was acquired by GBOX Chemi XT4 System and was finally quantified by Gene Tools software (Syngene, Cambridge, UK).

### Experiment *in vitro*

AML12 cells (mouse hepatocyte) were obtained from the Cell Biology Institute of Chinese Academy of Science (Shanghai, China) and cultured in DMEM/ Ham’s F-12 with 10% FBS (Lonsera, Grand Island, USA) at 37°C in an incubator under the atmosphere of 5% CO_2_. To mimic the HFD condition, cells were exposed to DMEM containing a 1 mM FFAs (oleate acid: palmitate acid=2:1, O/P) and 1% BSA (Sigma, Steinheim, Germany) for 24 h to induce steatosis. SNN (0.5μg/mL) and/or 1 μM Compound C (AMPK inhibitor) or 10 μM EX527 (SIRT1 inhibitor) (MedChemExpress, Shanghai, China) were added to the mixture of FFAs to incubate cells simultaneously. The dose of SNN was the best dose for improving AML12 cell viability determined by our previous tests. The concentration of AMPK or SIRT1 inhibitor was chosen according to the report of experiments *in vitro*. The cells cultured in DMEM containing 1% BSA were used as the control.

The lipid content in cells was determined using Nile Red (SIGMA) and DAPI (MP, Biomedicals, USA) staining. The cell images were scanned by ImageXpress Microsystem High-content imaging system. The lipid content in cells was quantified by ImageXpress Analysis (Molecular Devices).

### Statistical analysis

The data were expressed as mean ± standard deviation. SPSS 18.0 and Graphpad 8.0 were used for all statistical analyses and diagram drawings. Student *t*-test was used to compare the means of two groups. For three or more groups, statistical analyses were performed using one-way analysis of variance (ANOVA) followed by Dunnett’s post-hoc test. *P*<0.05 was considered statistically significant.

## Results

### Dynamic change of body weight, liver weight, serum biochemical indexes, and liver histopathology of mice fed with HFD

Table 3 shows the dynamic change of body weight, liver weight, serum level of ALT, AST, and lipid of mice fed with HFD. The body weight has been found higher in the model group since the end of the 4th week. The serological analysis demonstrated that, compared with a normal diet, HFD induced significantly higher levels of TC, HDL-c, and LDL-c since the end of the 8th week, but no obvious change in serum TG level. After 16 weeks, compared with the control, mice of the model group had significantly increased wet liver weight, body weight, serum ALT activity, and serum level of TC, HDL-c, and LDL-c.

**Table 3:** Dynamic change of body weight, liver weight, and serum biochemical indices of mice fed with HFD

Time	4W	8W			12W		16W	
Group	C	M	C	M	C	M	C	M
Body weight	23.73±0.93	30.93± 1.10^**^	27.67± 3.28	33.68± 1.08^*^	31± 2.26	38.8± 0.70^*^	31.8± 0.92	49.6± 1.09^***^
Liver weight	1.03± 0.07	0.75± 0.06	1.18± 0.16	1.20± 0.03	1.20± 0.01	1.30± 0.04	1.16± 0.11	1.64± 0.17^***^
Serum ALT	33.0± 3.0	32.17± 3.06	34.0± 2.29	29.17± 0.76	33.5± 11.63	35.67±5.49	29.0± 16.26	99.0± 10.04^**^
Serum AST	141.3±25.76	155.8± 46.2	164.0± 3.78	153.0± 40.3	162.7± 2.75	175.3±6.29	157.0±3.54	198.3± 24.06
Serum TG	0.82± 0.23	0.75± 0.18	1.93± 0.28	1.55± 0.49	1.00± 0.22	1.18± 0.21	1.43± 0.60	0.88± 0.15
Serum TC	1.90± 0.15	3.12± 0.25	2.60± 0.23	5.58± 0.08^***^	2.72± 0.40	5.63± 0.43^***^	3.28± 0.46	6.28± 0.84^***^
Serum HDL-c	1.87± 0.21	3.10± 0.23	2.52± 0.28	5.52± 0.24^***^	2.68± 0.43	5.60± 0.56^***^	3.58± 0.46	6.70± 0.69^***^
Serum LDL-c	0.23± 0.15	0.43± 0.15	0.12± 0.03	0.67± 0.15^*^	0.23± 0.06	0.85± 0.13	0.30± 0.14	1.15± 0.30^**^

Data are expressed as mean±SD, n=3 per group, ^***^P < 0.001, ^**^P < 0.01 compared to the control group

**Fig. 1:**
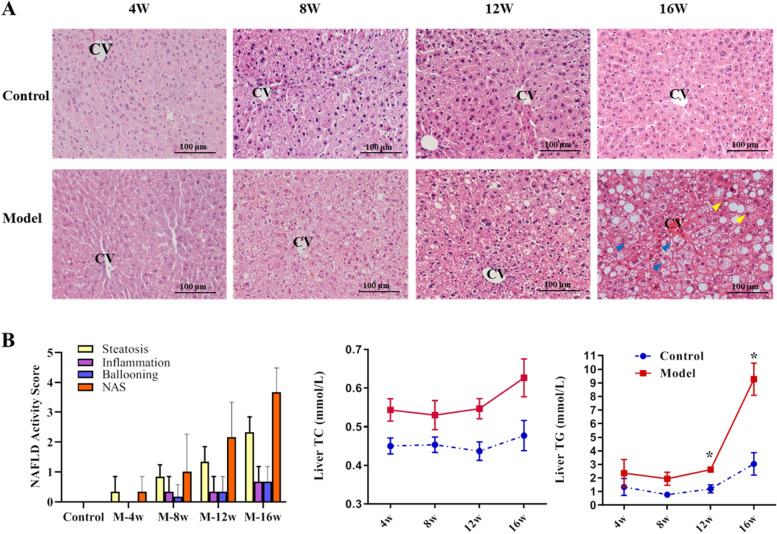
Dynamic change of liver histopathology and lipid content of mice fed with HFD. **A** HE staining of liver tissues; CV indicates lobular central vein; yellow arrows indicate inflammatory cells, blue arrows indicate ballooning degeneration; the original magnification is 200×. **B** Histological assessment of NAFLD activity score; **C** The dynamic change of liver TC and TG content. *n* = 3; *P < 0.05, vs. Control

HE staining and NAFLD activity score of liver tissues showed that no steatosis, inflammation, and hepatocyte ballooning were found in all of the control mice, the hepatosteatosis degree, and the apparent number of inflammatory infiltration and ballooning increased with the prolongation of HFD feeding time. At the end of the 16th week, there was extensive steatosis and many ballooning degenerations of hepatocytes appeared in the liver tissues of model mice, indicating the establishment of NAFLD (Fig. 1A, B). To further determine the dynamic change of lipid accumulation, TG and TC levels of liver tissues were determined (Fig. 1C). It was identified that hepatic TG and TC of the model group increased with the time of modeling, and TG of the model group was significantly higher than that of the control group after 12 and 16 weeks.

Taken together, these results suggest that HFD successfully induced mice NAFLD model, including elevated lipid levels of serum and liver and liver dysfunction.

### Dynamic changes of SIRT1 expression and AMPK activation in liver tissues of mice fed with HFD

**Fig. 2:**
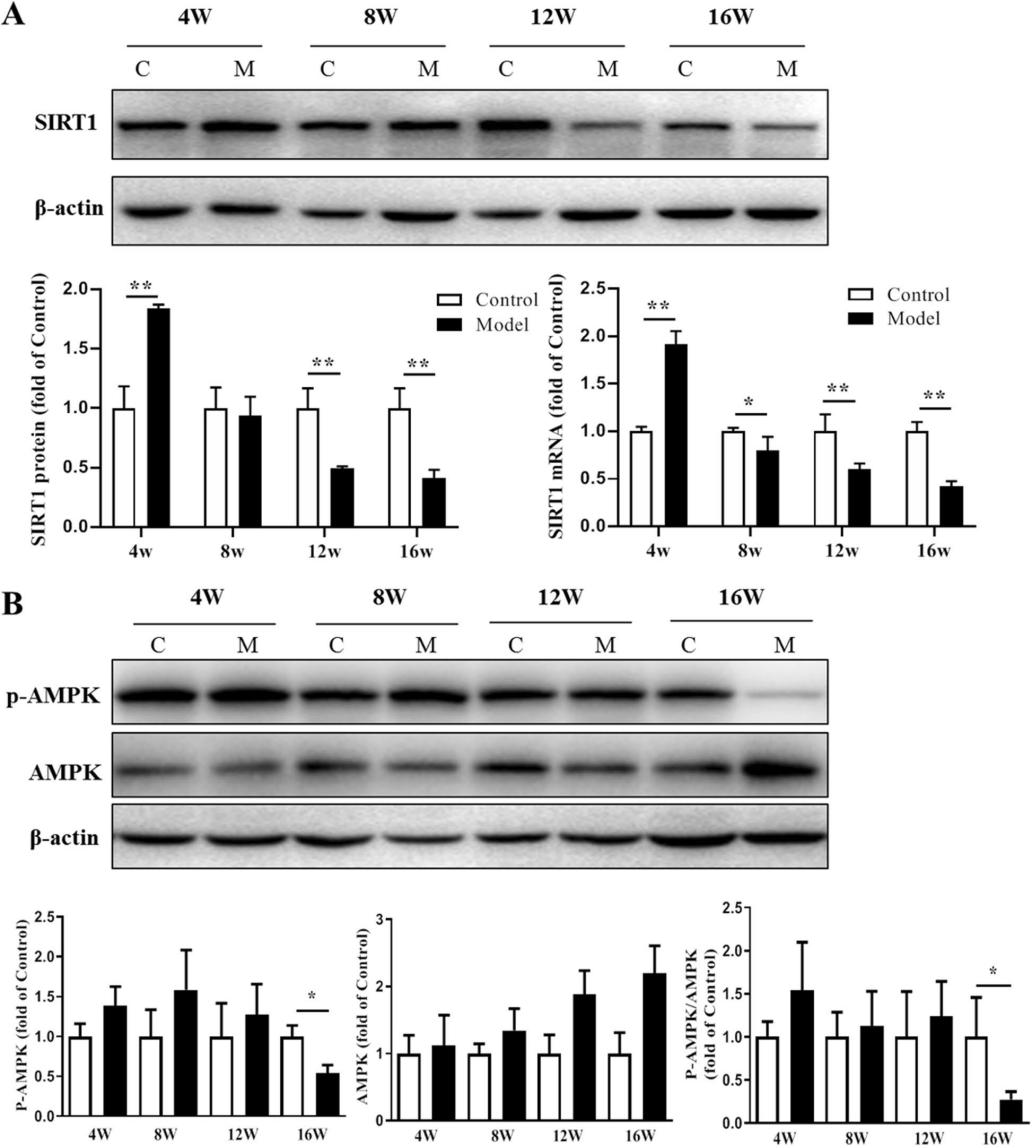
Dynamic change of SIRT1 expression and AMPK activation in liver tissues of mice. **A** The hepatic SIRT1 protein and mRNA expression level. **B** The hepatic expression level of AMPK and P-AMPK. *n* = 3; *P < 0.05, **P < 0.01 vs. Control

Compared with the control group, the protein and mRNA expression of SIRT1 in the model group was up-regulated at the end of the 4th week, but down-regulated since the 8th week (Fig. 2A). The protein expression of P-AMPK and the ratio of P-AMPK/AMPK in the model group showed an increasing trend versus the control from the 4th week to the 12th week, but no statistical significance possibly because of limited samples in each group. After 16 weeks, However, P-AMPK and P-AMPK/AMPK levels showed decreased in the model group than in the control group (Fig. 2B).

### SNN improved HFD-induced NAFLD in mice

**Fig. 3:**
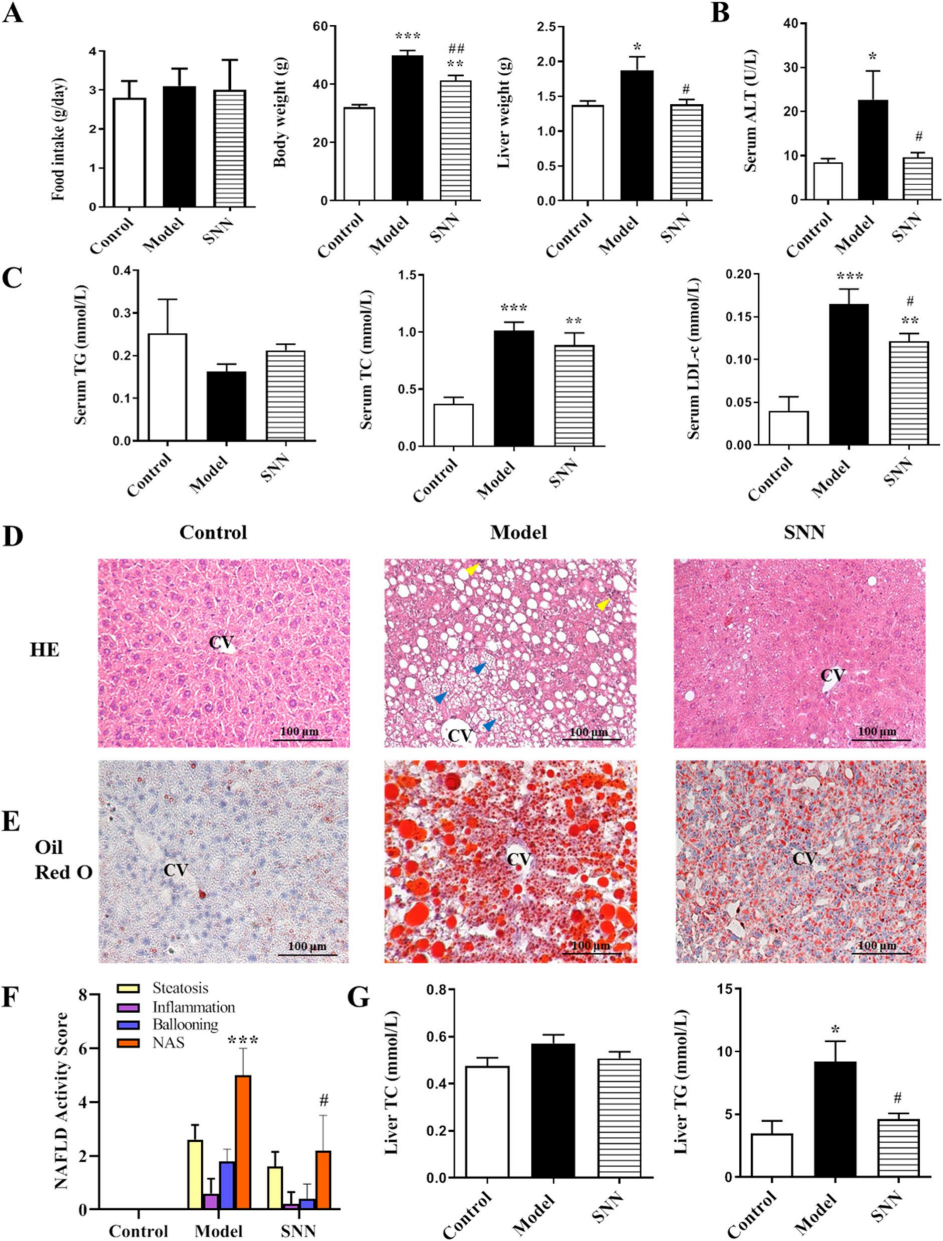
SNN improved HFD-induced mice NAFLD. **A** The body weight and liver weight. **B** The expression of serum ALT (**C**) serum TG, TC, and LDL-c. **D** HE staining and (**E**) Oil Red O staining of liver sections of mice; CV indicates lobular central vein; yellow arrows indicate inflammatory cells, blue arrows indicate ballooning degeneration; the original magnification is 200×. **F** Histological assessment of NAFLD activity score. **G** The content of liver TG and TC. *n* = 5-8; *P < 0.05, **P < 0.01, ***P < 0.001vs. Control; ^#^*P* < 0.05, ^##^*P* < 0.01 vs. Model

There was no difference among different groups found in the daily food intake for the last week of the experiment *in vivo*. In contrast to those of the control group, the body weight and liver weight remarkably increased in the model group. These parameters significantly improved in mice treated with SNN (Fig. 3A). Liver damage was exhibited by the elevated serum values of ALT in the model group which was down-regulated by SNN treatment (Fig. 3B). Statistically significant differences were also observed in the blood lipid content. The serum levels of TC and LDL-c increased in the model group, which were downregulated by SNN intervention (Fig. 3C). Figure 3D and E showed that the liver sections of the model mice stained with HE and Oil Red O displayed abundant accumulation of lipid droplets in hepatocytes, together with scattered inflammatory cell infiltration and hepatocellular ballooning degeneration, which were all improved in mice treated with SNN. The histological assessment according to the HE and Oil Red O staining also showed significantly higher NAS in the model mice than in the control and SNN group (Fig. 3F). The increased liver TG content, which was another proof of lipid deposition in the livers of model mice, was consistently reduced by SNN (*P*<0.05) (Fig. 3G).

### SNN reactivated the SIRT1/AMPK signaling pathway in liver tissues of NAFLD mice

**Fig. 4:**
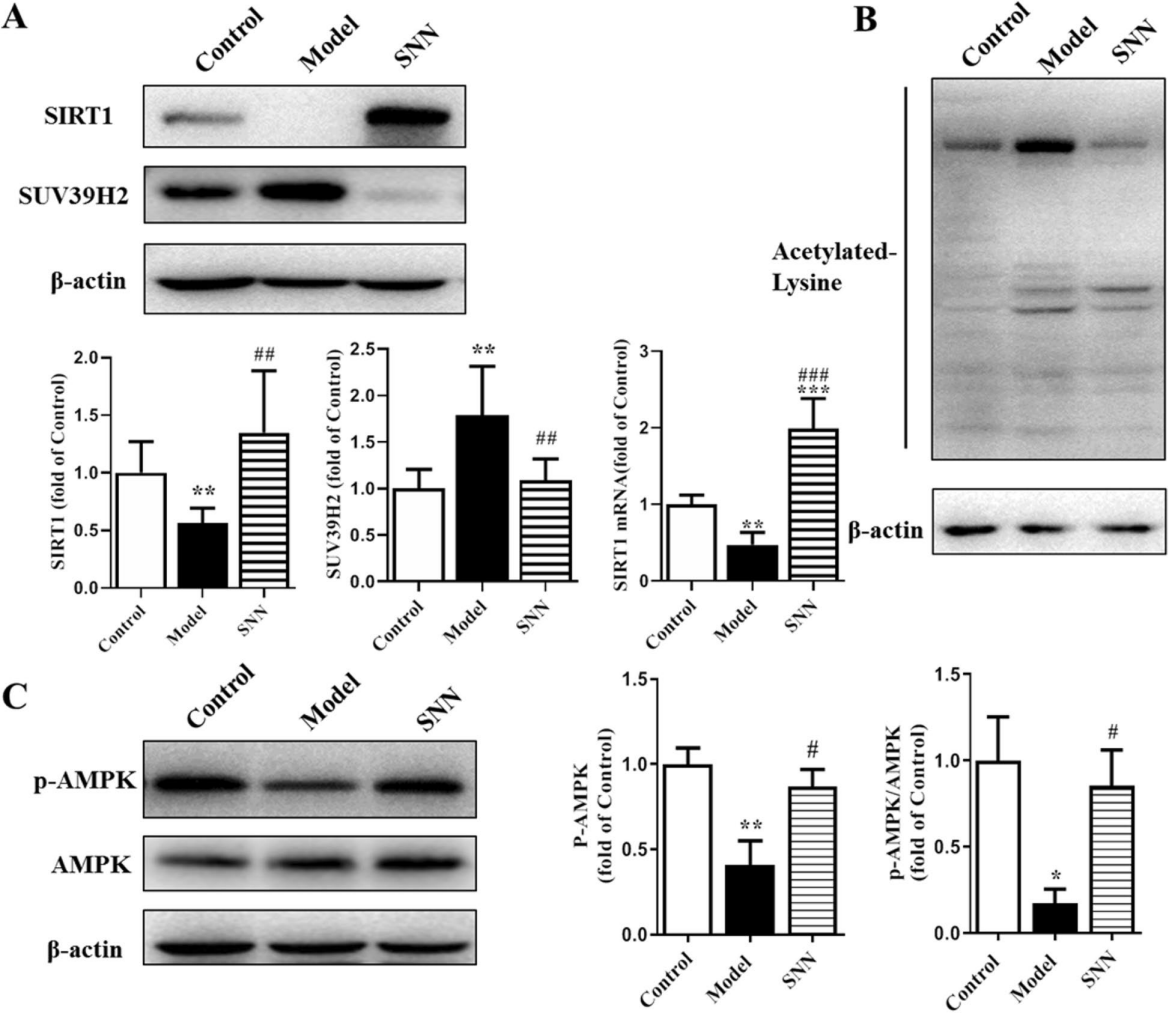
Hepatic expression of SIRT1 and activation of AMPK of mice. **A** Hepatic level of SIRT1 and SUV39H2 expression. **B** The level of acetylated-Lysine in liver tissues was evaluated by Western blot analysis. **C** The expression and activation of AMPK of mouse liver tissues. β-actin was determined as the loading control. *n* = 3-6; *P < 0.05, **P < 0.01, ***P < 0.001 vs. Control; ^#^*P* < 0.05, ^##^*P* < 0.01, ^###^*P* < 0.01vs. Model

To explore the underlying mechanism of the effect of SNN, the hepatic expression levels of molecules involved in the SIRT1/AMPK pathway were evaluated. Fig. 4A showed the Hepatic expression of SIRT1 was down-regulated in the NAFLD model group and was restored by SNN treatment. On the contrary, the negative regulatory gene of SIRT1, suppressor of variegation 39 homolog 2 (SUV39H2) was up-regulated in the model group and decreased by SNN intervention. SIRT1 played the role of de-acetylation. Figure 4B showed Acetylated-lysine increased in the model group and SNN down-regulated its level. HFD significantly suppressed the protein level of phospho-AMPKα. SNN treatment enhanced the phosphorylation of AMPKα and the ratio of P-AMPK/AMPK (Fig. 4C).

**Fig. 5:**
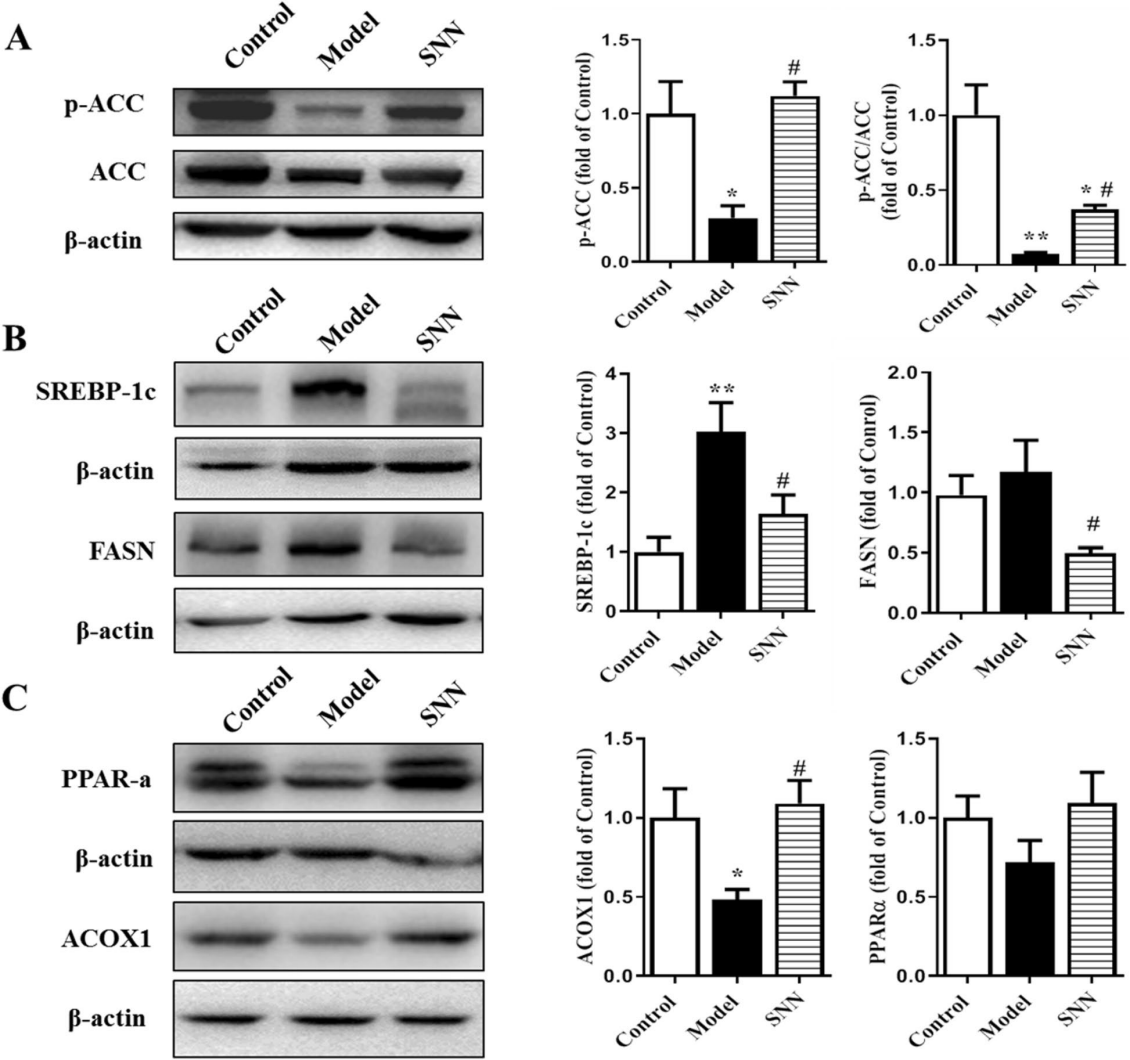
SNN regulated the hepatic expression of molecules associated with the lipid metabolism in NAFLD mice. **A** The level of ACC activation in liver tissues. **B** The hepatic protein expression of SREBP-1c, FASN. **C** The expression of PPARα and ACOX1 in liver tissues. β-actin was determined as the loading control. *n* = 3-6; *P < 0.05, **P < 0.01 vs. Control; ^#^*P* < 0.05 vs. Model

The expression levels of some downstream targets of the SIRT1/AMPK including ACC, FASN, SREBP-1c, ACOX1, and PPARα were also evaluated (Fig. 5). ACC is a key molecule in fatty acid synthesis and can inhibit fatty acid oxidation. Phosphorylation of ACC at Ser79 inactivated the molecule. HFD feeding significantly decreased the level of phospho-ACC (p-ACCSer79) and the ratio of P-ACC/ACC, which was up-regulated by SNN treatment. The results also showed the levels of SREBP-1c and FASN, the genes promoting fatty acid synthesis were increased in liver tissues of the model group. Additionally, the expression of ACOX1, the gene promoting oxidative degradation of fatty acid, was down-regulated in the model group. SNN treatment reduced the expression of SREBP-1c and FASN and increased the expression of ACOX1. The nuclear transcription factor PPARα, which can promote lipid oxidation, has the same trend as ACOX1.

### Inhibition of SIRT1 and AMPK attenuated the improvement of SNN on FFA-induced steatosis of hepatocytes

**Fig. 6:**
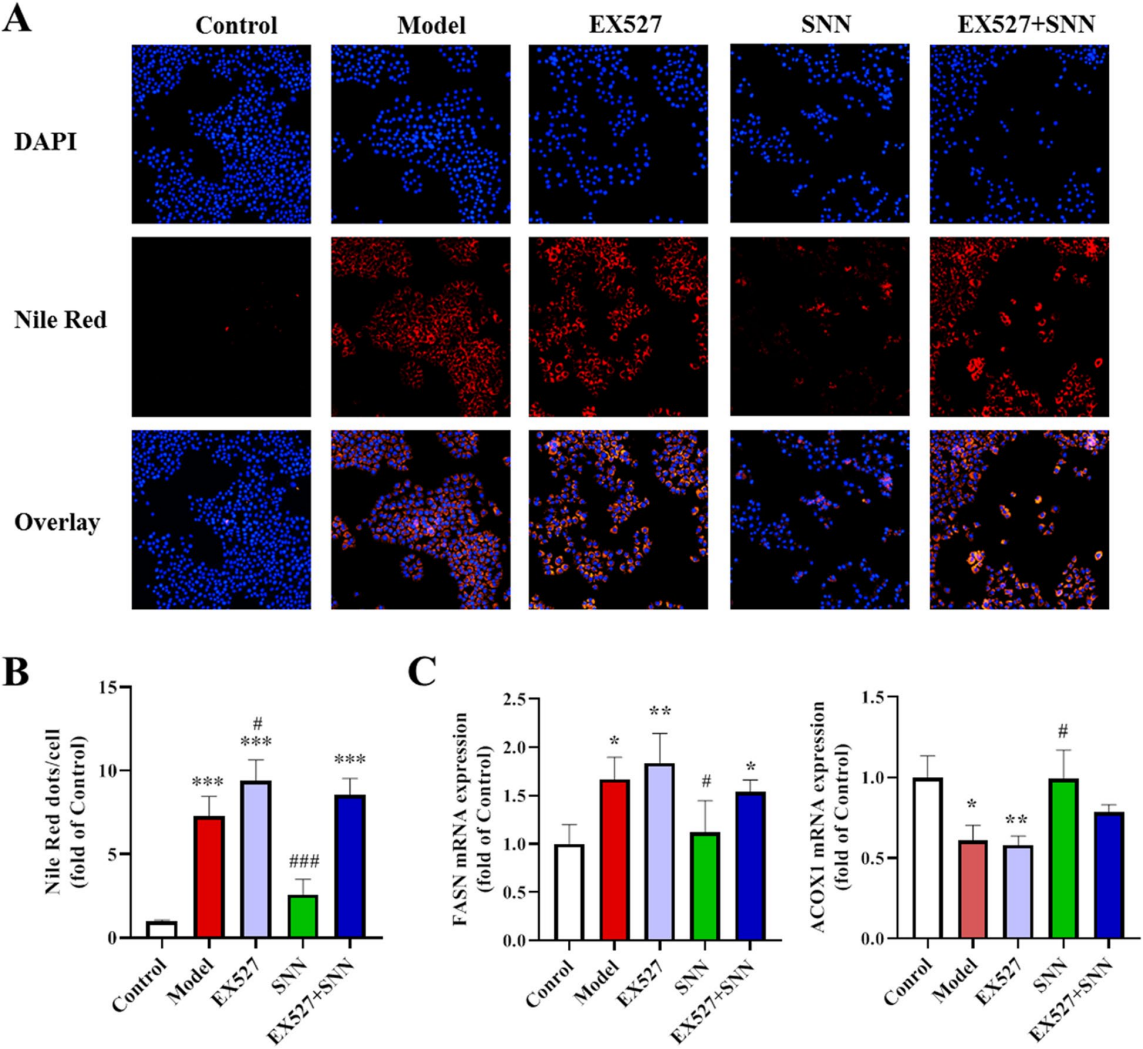
Inhibition of SIRT1 prevented the improvement of SNN on FFA-induced steatosis in AML12 cells. **A** DAPI and Nile Red double staining showed the level of lipid accumulation in AML12 cells (200×). **B** The quantification of Nile Red staining. **C** The mRNA level of molecules associated with lipid metabolism including FASN and ACOX1. *n* = 3-6; *P < 0.05, **P < 0.01, ***P < 0.001 vs. Control; ^#^*P* < 0.05, ^###^*P* < 0.01vs. Model

**Fig. 7:**
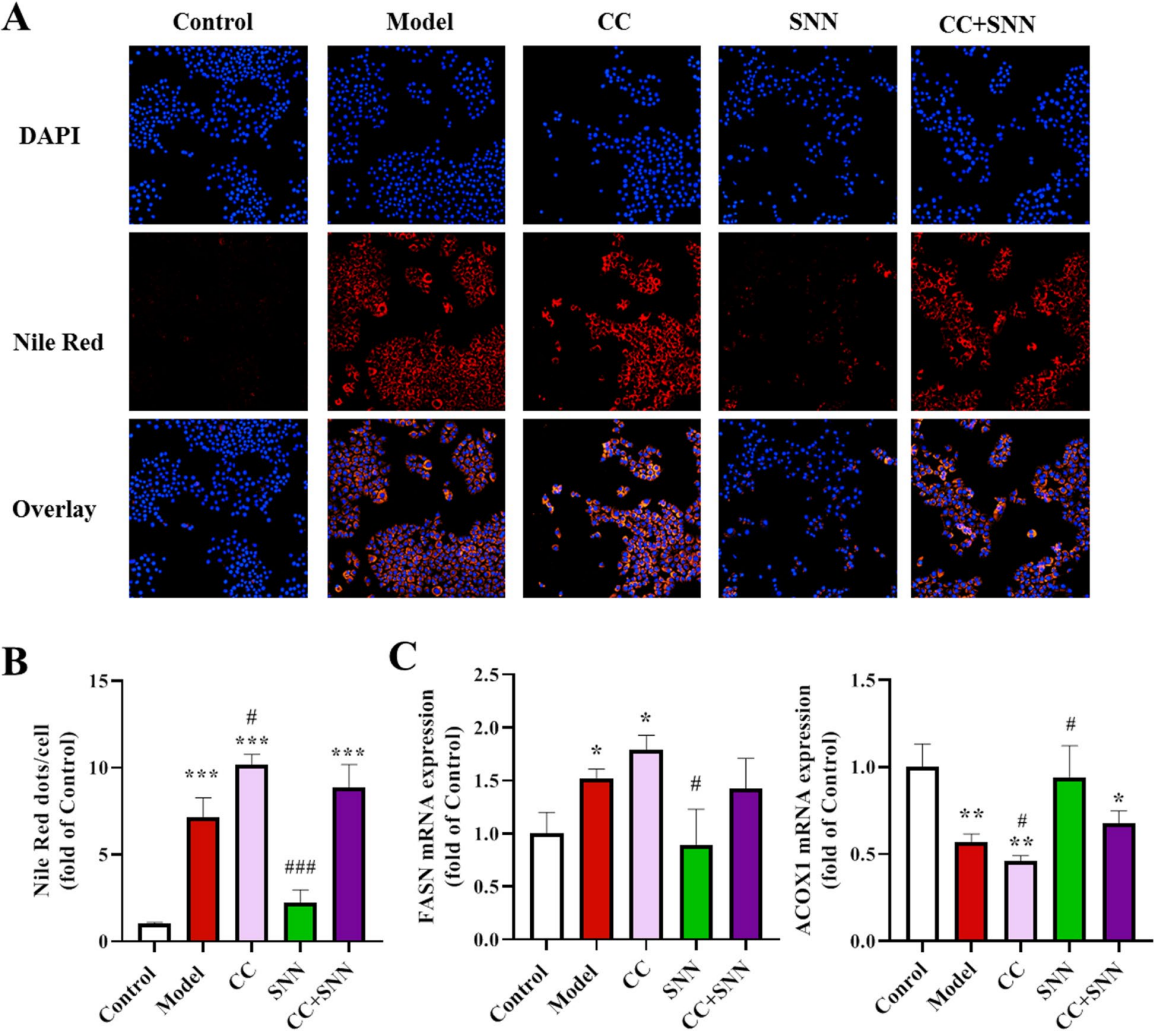
Inhibition of AMPK attenuated the improvement of SNN on FFA-induced steatosis in AML12 cells. **A** DAPI and Nile Red double staining showed the level of lipid accumulation in AML12 cells (200×). **B** The quantification of Nile Red staining. **C** The mRNA level of FASN and ACOX1. *n* = 3-6; *P < 0.05, **P < 0.01, ***P < 0.001 vs. Control; ^#^*P* < 0.05, ^###^*P* < 0.01vs. Model

To further clarify the effect and mechanism of SNN on NAFLD, *in vitro* experiments were performed by using AML12 cells induced by fatty acids to mimic the NAFLD *in vitro* model. The DAPI staining of cells showed a large number of lipid droplets in cells induced by fatty acids, which was enhanced by the inhibitor of SIRT1 (EX527) or AMPK (Compound C). Treatment with SNN significantly ameliorated hepatic lipid accumulation. However, the anti-steatosis effect was diminished by EX527 or Compound C (Fig. 6A-B and 7A-B). FASN was found up-regulated in OA/PA-incubated cells compared with the control, and this increase was suppressed by SNN co-incubation. In contrast, ACOX1 expression was shown downregulated in the model group, which was shown elevated by SNN treatment. Consistently, as the downstream genes of AMPK and SIRT1, the expression levels of FASN and ACOX were regulated by the inhibitor of AMPK and SIRT1. EX527 or Compound C application almost blocked the regulated effect of SNN on the lipid metabolism-related genes (Fig. 6C and 7C). These results suggest that SNN could ameliorate lipogenesis and promote the oxidation of fatty acid through the AMPK/SIRT1signaling pathway.

## Discussion

HFD-induced mouse or rat model is commonly used for the research of pathogenesis and drug development of NAFLD due to its similar pathogenesis with human NAFLD. In this study, male C57BL/6J mice fed with HFD were used to establish the NAFLD model. changes in serum biochemical indexes and liver pathology were after the 4th, 8th, 12th, and 16th week, respectively. The dynamic monitored results showed that, with the time elongation, the body weight, liver weight, serum lipid, and transaminases of HFD-fed mice increased more seriously than those of the control mice. It has been reported that the prevalence of NAFLD is up to 50% in people with dyslipidemia. The serum levels of TC and LDL-c presented in this model were similar to the increase of serum cholesterol in people with NAFLD, which is closely related to NAFLD [22]. However, there is no obvious difference found in the serum TG between model and control mice for all the timepoint. This result was similar to our previous experimental result, demonstrating no change in serum TG level in the mice fed with HFD for 18 weeks [23, 24]. Another study for the NAFLD model also showed mild downregulated trend of serum TG in C57BL/6, CD-1, and 129Sv mice after HFD feeding for 9 weeks [25]. Elevated serum ALT indicating liver damage was also found in the model mice. More importantly, the liver steatosis area and degree of mice gradually aggravated with longer HFD, and at the end of the 16th week, slight inflammation and ballooning degeneration of hepatocytes even appeared in the liver tissue of model mice. Taken together, after a 16-week HFD, the mice became obese with hypercholesterolemia, hepatic lipid accumulation, and liver injury, confirming the development of NAFLD, which is consistent with the previously reported studies [26, 27]. Further, after another 6 weeks, the aforementioned indices increased more seriously at the end of the 22nd week of the experiment.

Using the HFD-induced NAFLD mice, the effect of SNN was investigated. The mice with SNN administration had lower body weight, liver weight, serum LDL-c, and ALT than those of the NAFLD model. In addition, hepatosteatosis, inflammatory cell infiltration, and ballooning in the liver tissues of model mice were significantly reduced by SNN. Consistently, the intrahepatic TG content was downregulated by SNN. These results suggested that SNN can ameliorate the lipid accumulation and liver damage of the NAFLD mice, which is consistent with studies using other HFD-induced NAFLD rodent models [13, 14, 16].

SIRT1 is an NAD^+^-dependent deacetylase, which can promote the deacetylation of lysine residues of various proteins. A large number of recent studies revealed that SIRT1 regulates lipid homeostasis through multiple nutrient sensors such as SREBP-1, AMPK, PGC1α, and PPARα [28–31]. SIRT1 can enhance insulin sensitivity, regulate liver lipid metabolism, suppress oxidative stress, and reduce the inflammatory response. Studies have shown that decreased expression and/or activity of SIRT1 plays a key role in NAFLD development [32–34]. Moreover, SIRT1 is negatively related to NAFLD degree. Its plasma values were found lower in severe NAFLD compared to simple mild hepatosteatosis [35]. Many factors may contribute to the down-regulation of the SIRT1 level. NAFLD hepatic tissues and FFA-treated HepG2 and Huh-7 cells presented miR-122 upregulation, which was found able to suppress Sirt1 expression via binding to its 3′-untranslated region (UTR) [36]. SUV39H2 expression could be induced by pro-NASH stimuli in the hepatocyte, mice, and human livers. It was found able to bind to the SIRT1 gene promoter and suppress SIRT1 transcription [37]. On the other hand, SIRT1 could be interacted with and subsequently degraded through ubiquitination by the gene related to energy in lymphocytes (GRAIL), which was upregulated in the livers of humans and mice with hepatic steatosis [38]. In the present study, 4-week HFD resulted in a higher hepatic level of SIRT1 than control mice, however, since the 8th week of a high-fat diet, the expression of this molecule in the liver of model mice was significantly lower than that of control mice. Besides the decreased expression of SIRT1, we also found increased total Acetylated-lysine in NAFLD mice, indicating less de-acetylation activity of SIRT1. Similarly, our result also showed that SUV39H2 was up-regulated in the mice liver of the NAFLD model group. The SNN treatment increased the expression level of SIRT1, but decreased the Acetylated-lysine and SUV39H2, suggesting SNN may upregulate SIRT1 expression by inhibiting SUV39H2.

AMPK is a metabolic sensor, composed of a catalytic (α) subunit and two regulatory (β, γ) subunits, and AMPK activation requires phosphorylation of the α subunit [39, 40]. AMPK is the major negative kinase regulator of ACC through phosphorylation to inhibit ACC activity. The latter molecule plays an essential role in inhibiting fatty acid oxidation through inhibiting carnitine palmitoyltransferase (CPT) and promoting fatty acid synthesis [41]. Studies have also shown that AMPK could down-regulate the expression and maturation of SREBP-1 [42], thus reducing the expression level of FASN and stearyl coenzyme A dehydrogenase-1 (SCD-1), thereby inhibiting the synthesis of fatty acids and triglycerides. AMPK can enhance the activity of PPARα, thereby increasing the expression of some key enzymes of lipid oxidation including ACOX, which plays a key role in promoting lipid oxidation degradation [43]. AMPK activation can inhibit the synthesis of de novo lipid and promote the oxidation of fatty acids. AMPK activity decreases in NAFLD, which can serve as an important target for treatment [44]. In our study, it was identified that AMPK activation had a higher trend in the liver tissues of mice with HFD feeding from the 4th week, but decreased with NAFLD progress. And the obvious difference was displayed between the model and control at the 16th week and the end of the experiment (22 w). The SNN treatment significantly upregulated the AMPK activation.

Both SIRT1 and AMPK were known to regulate each other and share many common target molecules, and the interaction between SIRT1 and AMPK could be reciprocal [45]. AMPK can increase the activity of SIRT1 by increasing the level of NAD^+^ [46]. SIRT1 activity relies on AMPK and was also shown to regulate AMPK activation in NAFLD [9, 10]. SIRT1 can activate AMPK by deacetylating its upstream regulator-LKB1 [47]. AMPK and SIRT1 have synergistic effects on energy metabolism and affect the pathogenesis of NAFLD. The suppression of SIRT1/AMPK resulted in reduced fatty acid utilization and abnormal lipid deposition in the liver [6, 45, 48]. In the present study, compared with control, the hepatic protein expression level of SREBP-1c was significantly increased, while the levels of p-ACC and ACOX1 decreased in the model group. SNN treatment downregulated SREBP-1c and FASN and upregulated p-ACC and ACOX1. There was an increasing trend of PPARα levels in the liver of mice with SNN treatment. These results *in vivo* suggest that SNN may affect lipid metabolism via regulating SIRT1/AMPK signaling. This was further verified by the *in vitro* experiments. SNN downregulated the lipogenesis gene and upregulated the gene for oxidation in AML-12 cells with FFA incubaton. However, the inhibition of SIRT1 or AMPK significantly diminished these effects and attenuated the improvement of SNN on FFA-induced steatosis of the hepatocytes. Therefore, the effect of SNN on the lipid metabolism of NAFLD is SIRT1/AMPK dependent.

A series of recent studies have shown that the effects of the active components in TCM herbs on NAFLD are associated with activating the SIRT1/AMPK pathway [49]. As one TCM extract, SNN contains a variety of compounds, some of which have been found to regulate lipid metabolism through the SIRT1/AMPK signaling pathway. *In vitro* and animal model studies have shown resveratrol reduced the hepatic accumulation of lipids and improves lipid and glucose metabolism. And evidence support that resveratrol activates SIRT1 via activation of AMPK, thereby inducing the deacetylation of SIRT1 targets, PGC-1a and FOXO1 [50]. Polydatin was proved able to prevent NASH via inhibition of oxidative stress and inflammation, as well as via regulation of multiple signaling pathways, including AMPK/LDLR, LKB1/AMPK, SIRT1-PGC-1α, etc [51]. One study demonstrated that emodin effectively ameliorated hepatic steatosis through the CaMKK-AMPK-mTOR-p70S6K-SREBP1 signaling pathway [52]. Another study also find that emodin was closely related to the regulation of AMPK signaling pathway which increases IR and fatty acid oxidation [53]. Accumulating evidence obtained from animal experiments proves that quercetin has beneficial effects on metabolism diseases. One study found a direct anti-lipogenic effect of quercetin exerted by inhibiting the de novo lipid (DNL) pathway by acting on the ACACA/AMPK/PP2A axis [54]. Resveratrol, polydatin, emodin and quercetin are the major components of SNN identified in mice serum and liver. Although many preclinical trials have shown the promising potential of these natural compounds against NALFD, however, corresponding human clinical studies on their effects remain scarce. But these research results suggest that the bioactive compounds such as resveratrol, polydatin, emodin, etc. in SSN may contribute to the therapeutic effects of SNN on NAFLD through the SIRT1/AMPK signaling pathway.

## Conclusions

In summary, our findings demonstrate that the abnormality of the SIRT1/AMPK signaling pathway is involved in the dysfunction of lipid synthesis and degradation during NAFLD development. SNN administration could improve the lipid metabolism disorder and liver damage of NAFLD, and the regulation of the SIRT1/AMPK signaling pathway may contribute to the underlying mechanism. These results are beneficial to comprehending the therapeutic effect of the traditional herbal formula and further promote its clinical application.

## Supplementary Information

**Additional file 1.**

## Data Availability

The datasets used and/or analyzed during the current study are available from the corresponding author on reasonable request.

## Abbreviations

ACC1: acetyl-CoA carboxylase 1
ALT: almandine Aminotransferase
AMPK: AMP-activated protein kinase
ACOX: acyl-Coenzyme A oxidase
AST: aspartate Aminotransferase
FASN: fatty acid synthase
FFA: free fatty acid
HDL-c: high-density lipid cholesterol
HE: hematoxylin-eosin
HFD: high-fat diet
LDL-c: low-density lipid cholesterol
NAFLD: nonalcoholic fatty liver disease
NAS: NAFLD activity score
NASH: nonalcoholic steatohepatitis
PCR: polymerase chain reaction
PPAR-α: peroxisome proliferator activated receptor-α
RT-PCR: reverse transcription-polymerase chain reaction
SREBP-1c: sterol regulatory element-binding protein-1c
SIRT1: silent information regulator 1
SUV39H2: suppressor of variegation 39 homolog 2
TC: total cholesterol
TCM: traditional Chinese medicine
TG: triglyceride
