# Supplementary material for: Salvia-Nelumbinis naturalis improves lipid metabolism of NAFLD by regulating the SIRT1/AMPK signaling pathway
Source: BMC Complement Med Ther. 2022 Aug 9;22:213. doi: 10.1186/s12906-022-03697-9 (PMC9361555; doi:10.1186/s12906-022-03697-9)
Supplement: Supplementary file 1 — Additional file 1. [file 12906_2022_3697_MOESM1_ESM.pdf]

Figure 2A.

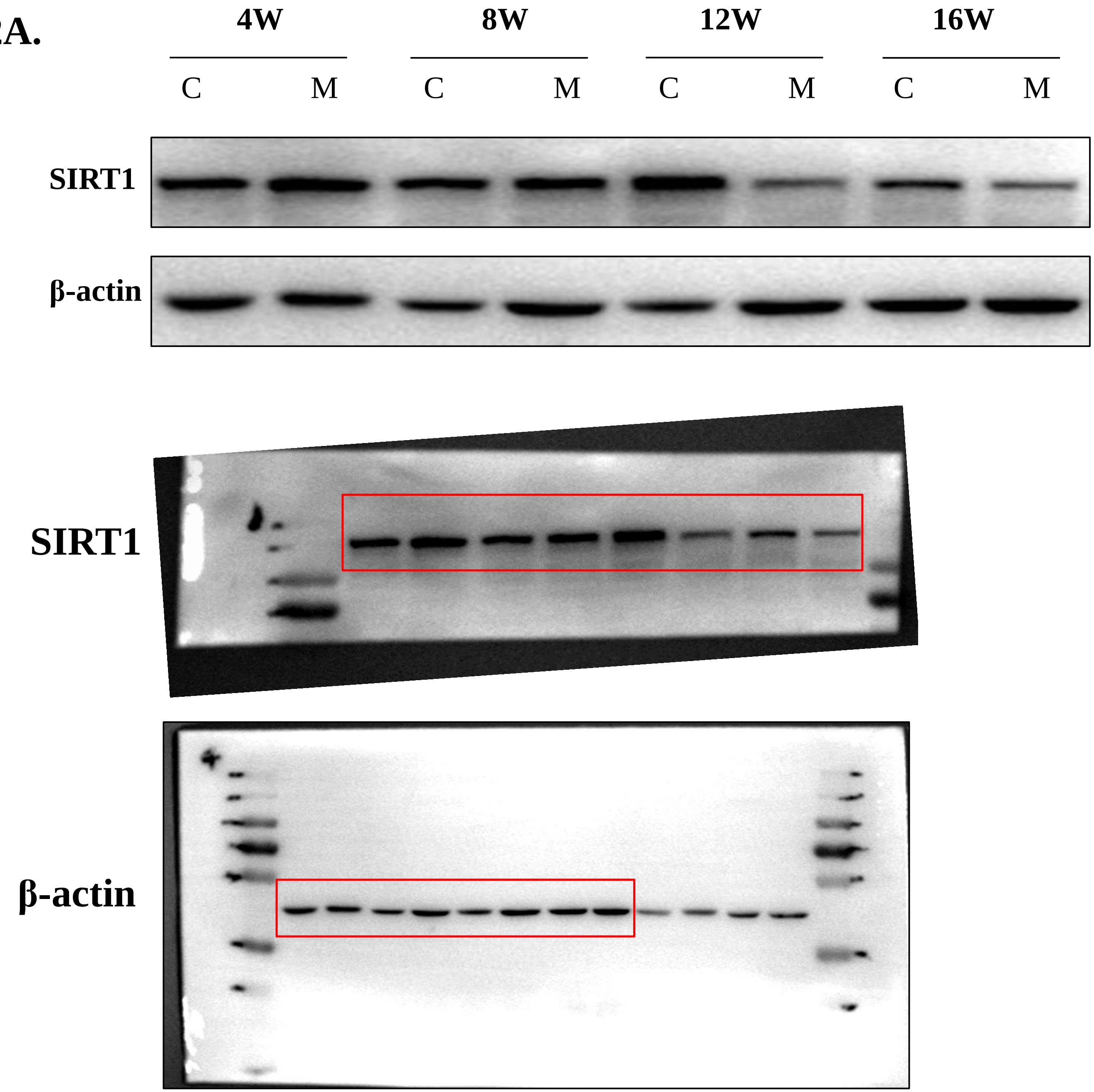

Figure 2B.

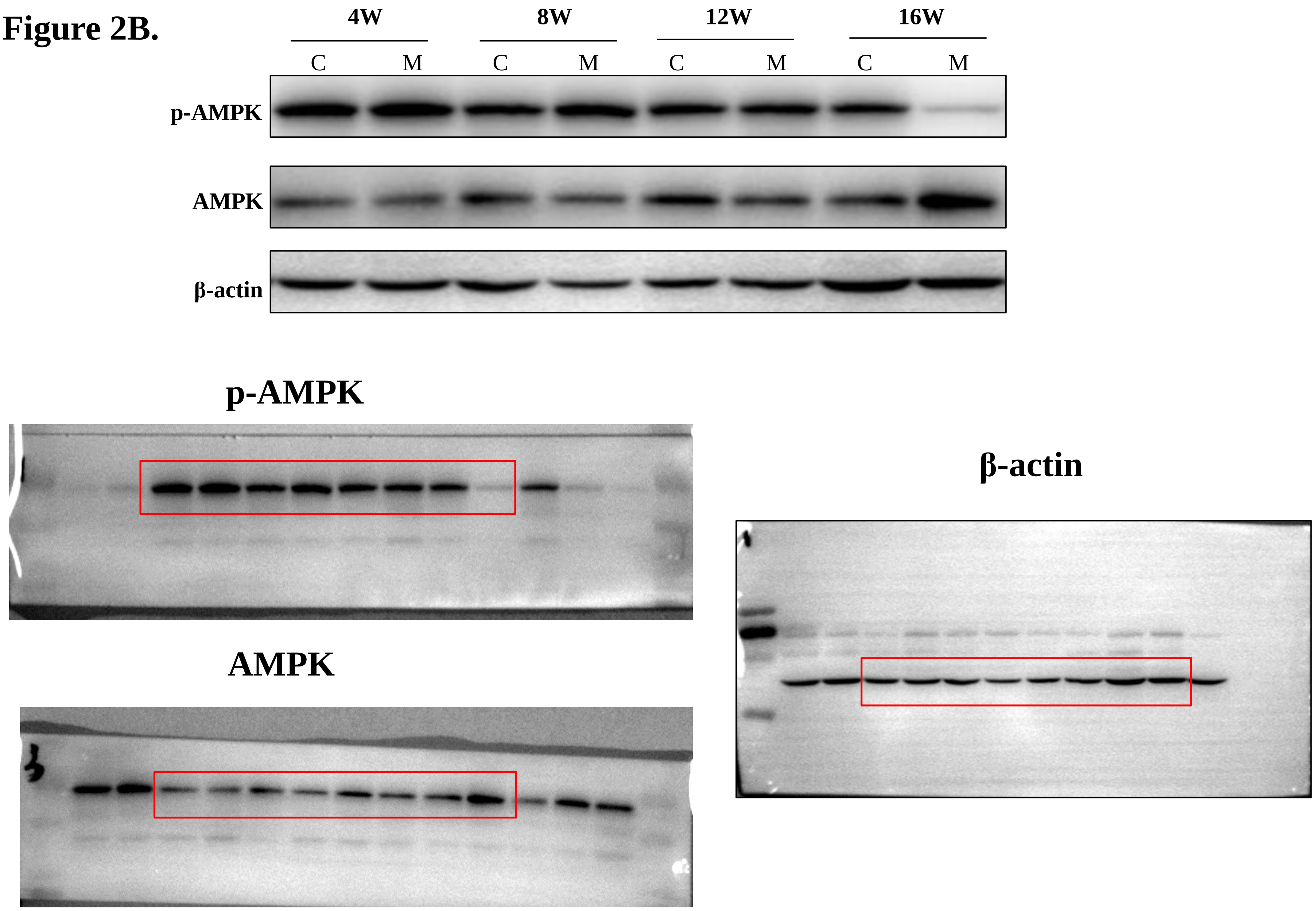

Figure 4A.

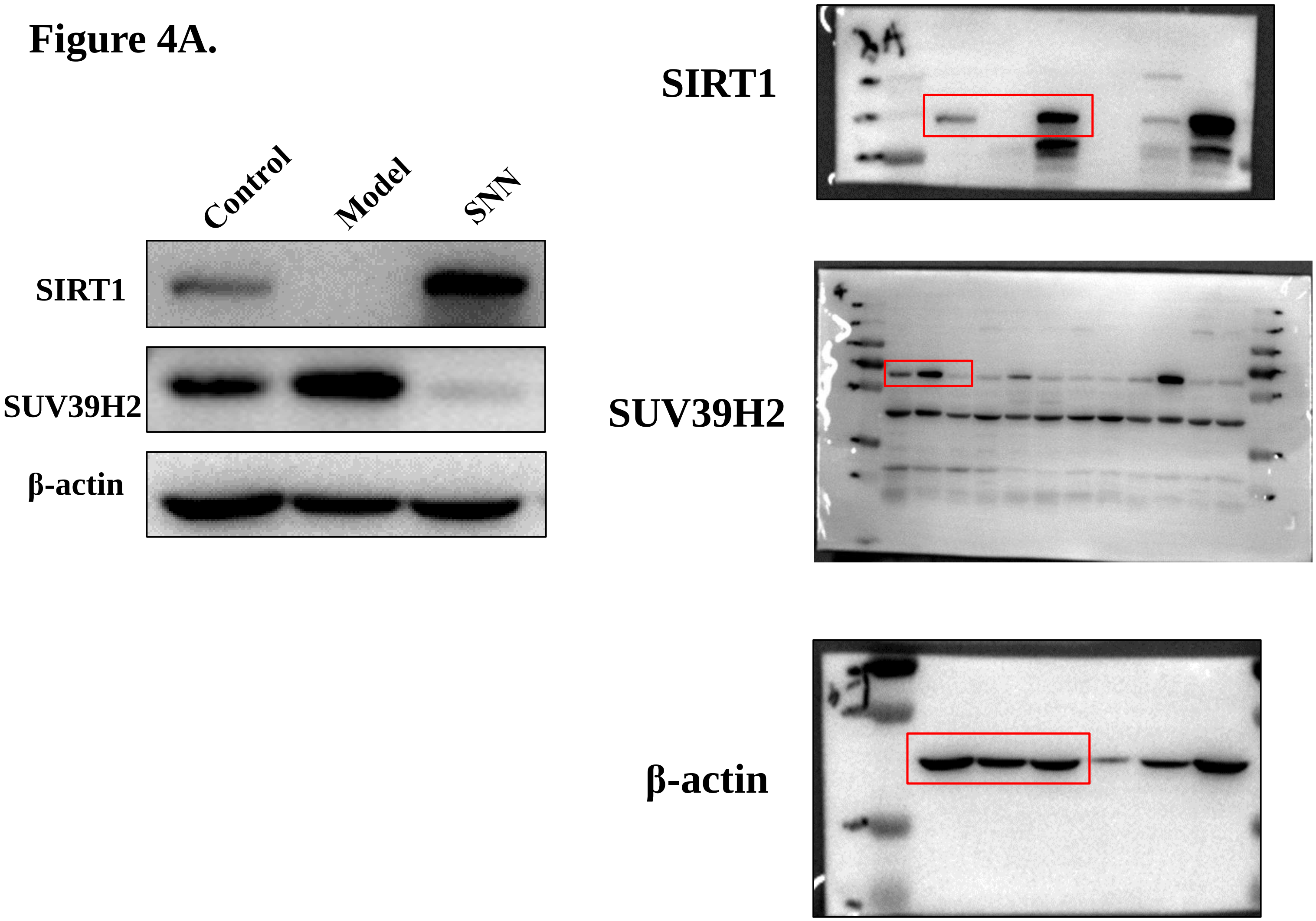
Control
Model
SNN
SIRT1
SUV39H2
β-actin
SIRT1
SUV39H2
β-actin

Figure 4B.

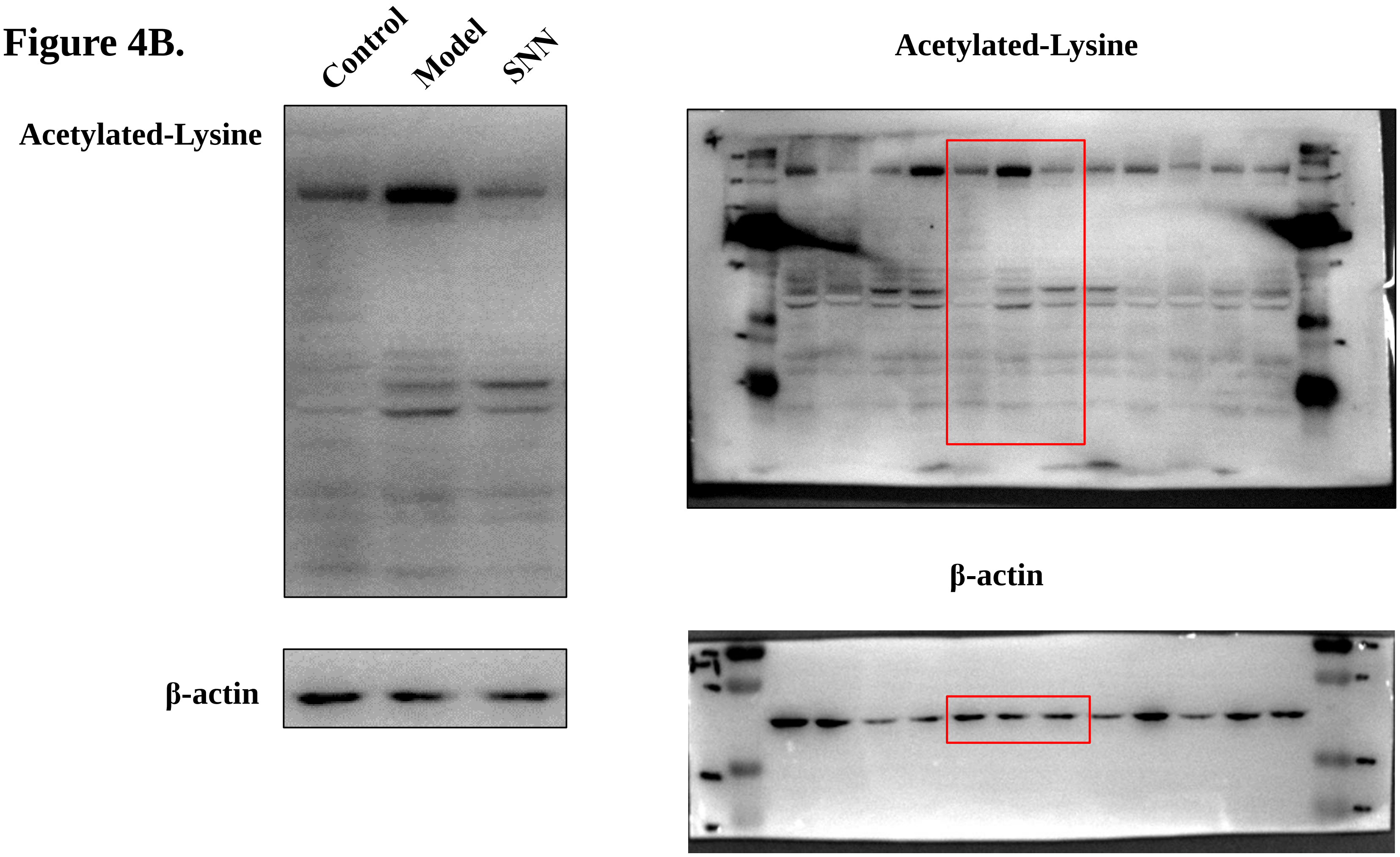

Control
Model
SNN
Acetylated-Lysine
β-actin
Acetylated-Lysine
β-actin

Figure 4C.

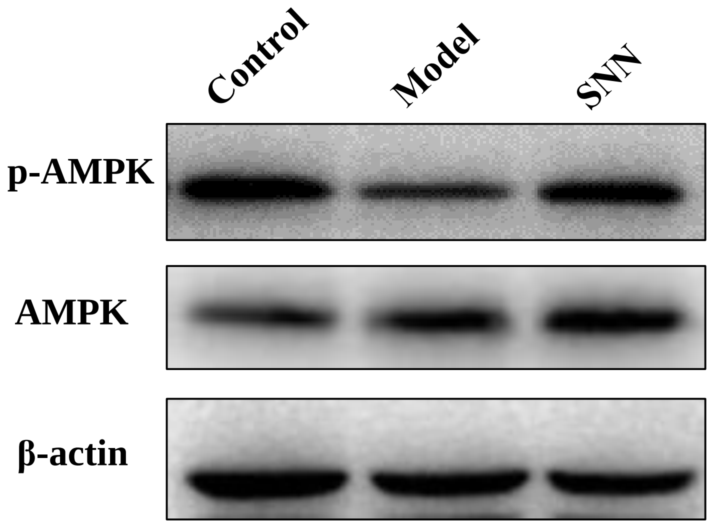

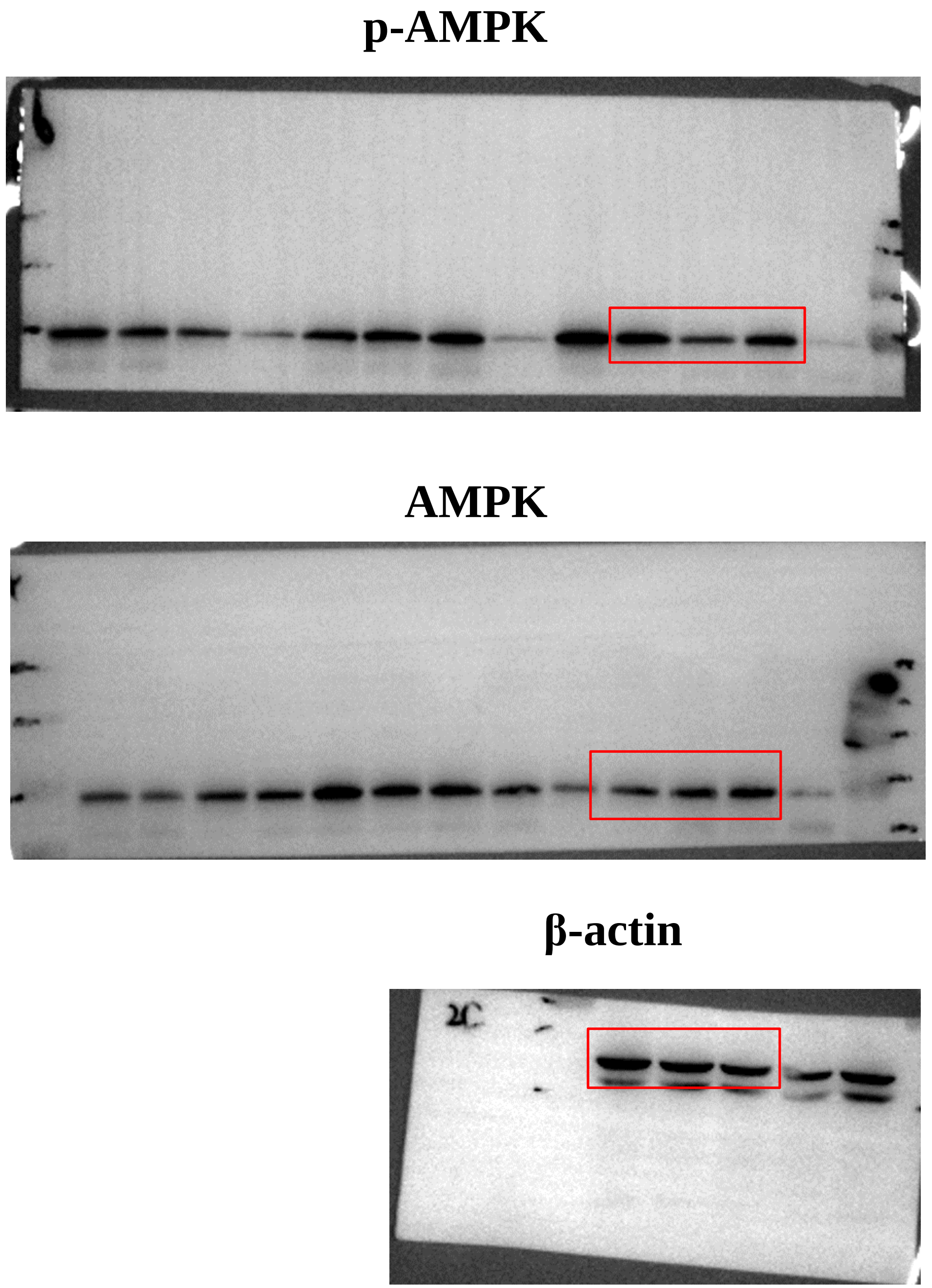

Figure 5A.

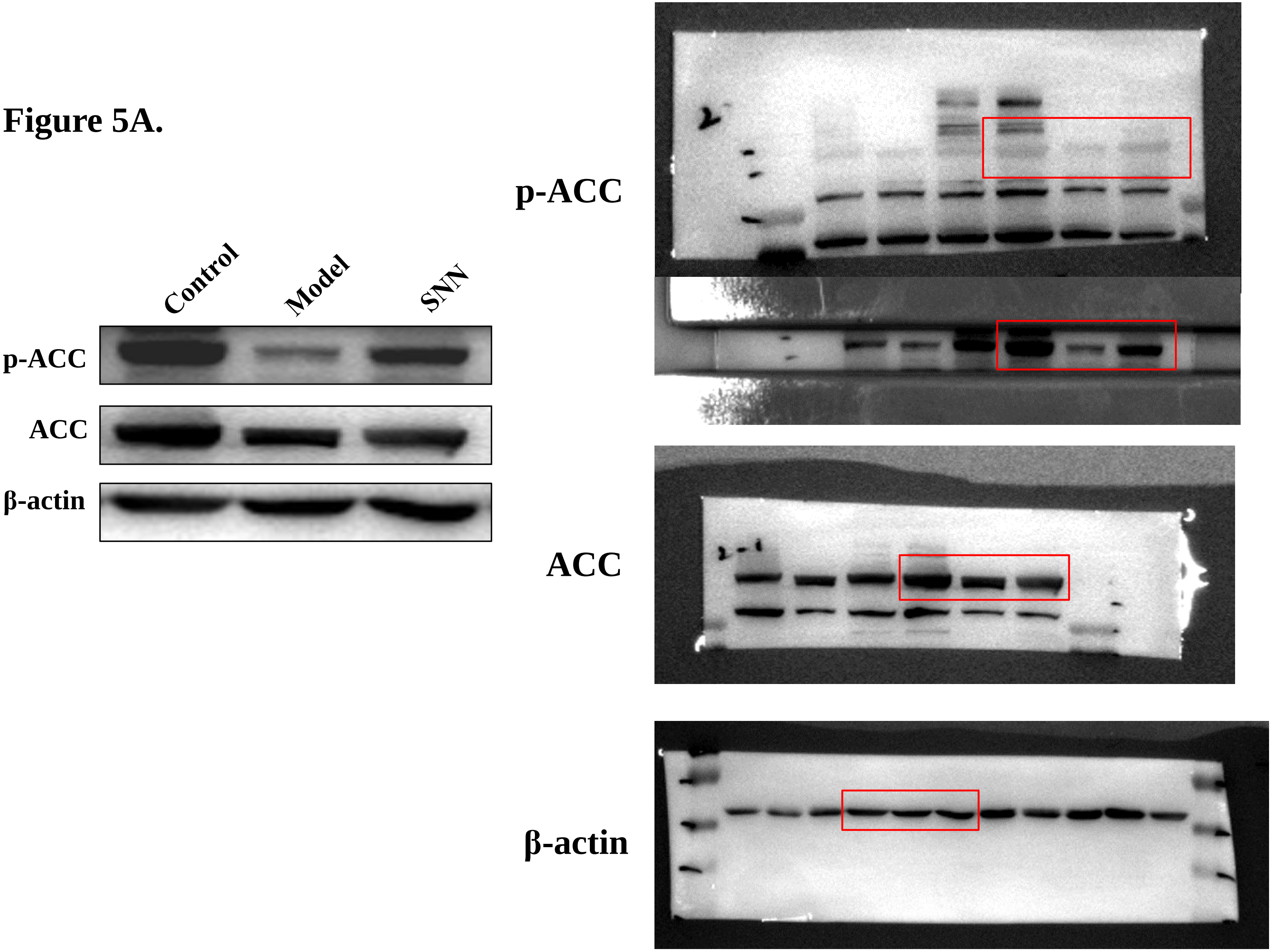

Figure 5B.

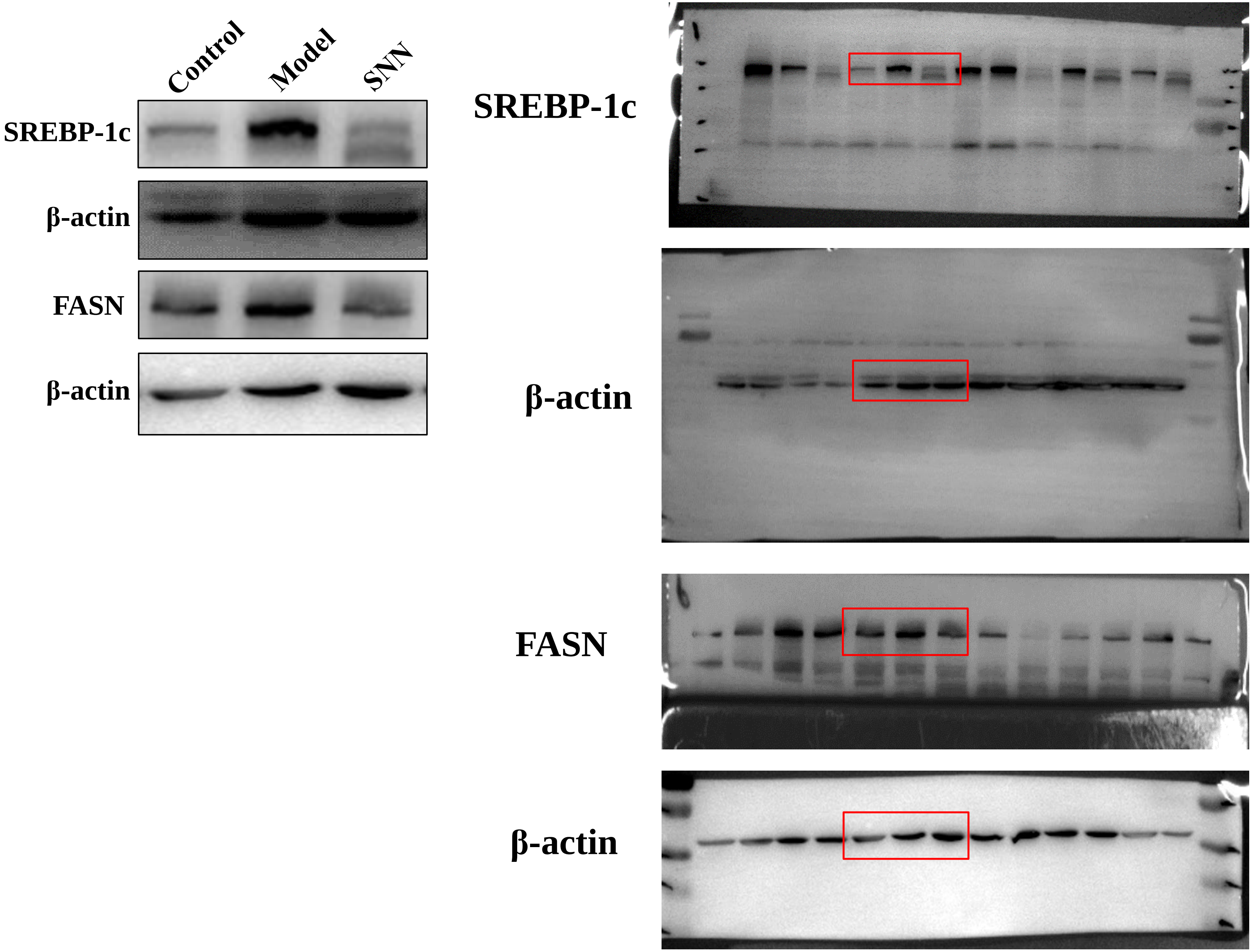
Control
Model
SNN
SREBP-1c
β-actin
FASN
β-actin
SREBP-1c
β-actin
FASN
β-actin

Figure 5D.

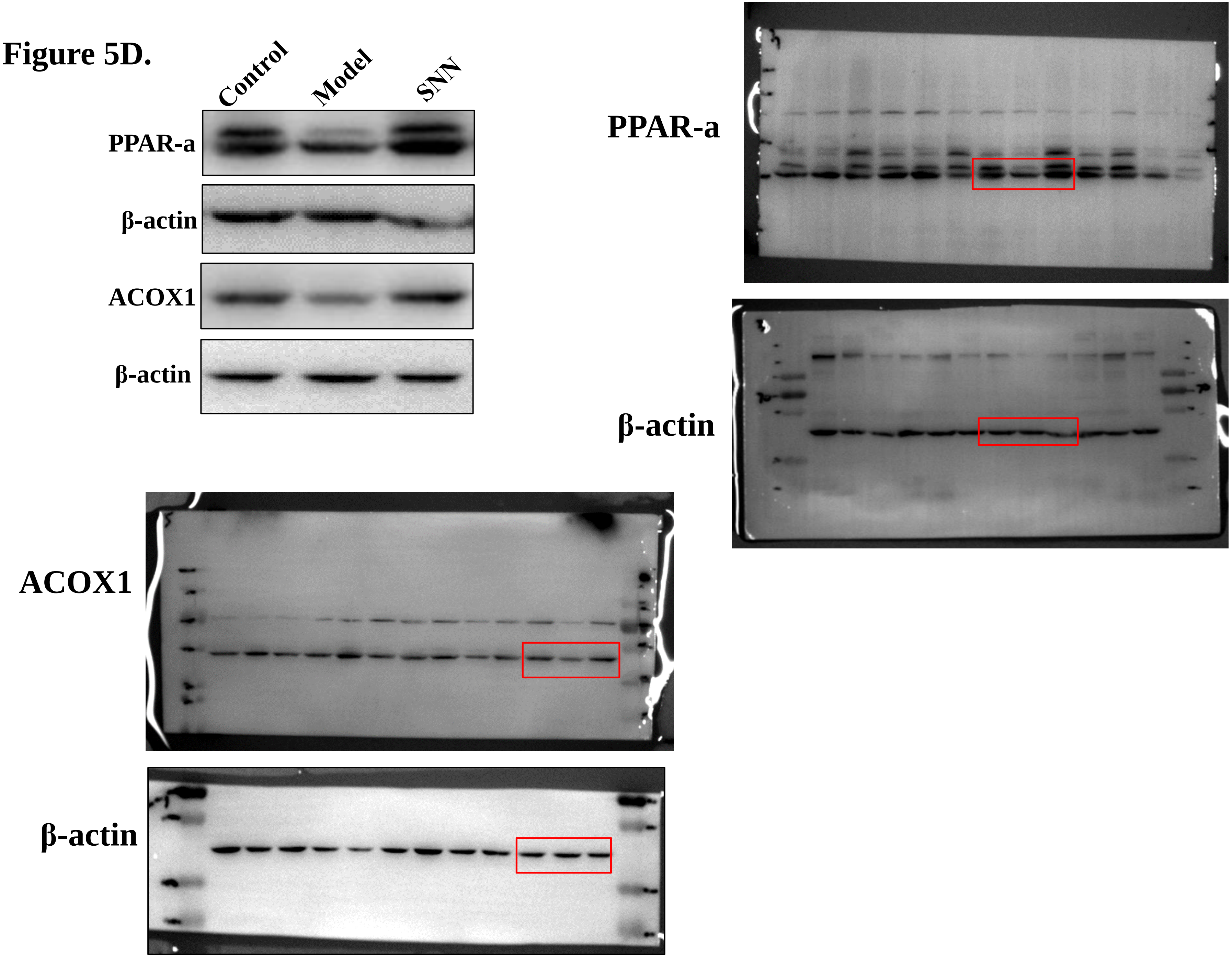
Control
Model
SNN
PPAR-a
β-actin
ACOX1
β-actin
PPAR-a
β-actin
ACOX1
β-actin
